# Rock climbing activity and physical habitat attributes impact avian community diversity in cliff environments

**DOI:** 10.1371/journal.pone.0209557

**Published:** 2019-01-16

**Authors:** Nora Covy, Lauryn Benedict, William H. Keeley

**Affiliations:** 1 School of Biological Sciences, University of Northern Colorado, Greeley, CO, United States of America; 2 City of Boulder Open Space and Mountain Parks, Boulder, CO, United States of America; University of Lleida, SPAIN

## Abstract

As the sport of outdoor rock climbing rapidly grows, there is increasing pressure to understand how it can affect communities of organisms in cliff habitats. To that end, we surveyed 32 cliff sites in Boulder, Colorado, USA, and assessed the relative roles of human recreation and natural habitat features as drivers of bird diversity and activity. We detected only native avian species during our observations. Whereas avian abundance was not affected by climbing, avian species diversity and community conservation value were higher at low-use climbing formations. Models indicated that climber presence and cliff aspect were important predictors of both avian diversity and avian cliff use within our study area, while long-term climbing use frequency has a smaller, but still negative association with conservation value and cliff use by birds in the area. In contrast, the diversity of species on the cliff itself was not affected by any of our measured factors. To assess additional community dynamics, we surveyed vegetation and arthropods at ten site pairs. Climbing negatively affected lichen communities, but did not significantly affect other vegetation metrics or arthropods. We found no correlations between avian diversity and diversity of either vegetation or arthropods. Avian cliff use rate was positively correlated with arthropod biomass. We conclude that while rock climbing is associated with lower community diversity at cliffs, some common cliff-dwelling birds, arthropods and plants appear to be tolerant of climbing activity. An abiotic factor, cliff aspect strongly affected patterns of both avian diversity and cliff use, suggesting that the negative effects of rock climbing may be mitigated by informed management of cliff habitat that considers multiple site features.

## Introduction

Cliff ecosystems serve as refuges for organisms, including many birds [1], and support unique species and diverse communities [2, 3]. Cliffs provide protection from traditional disturbance pressures, such as trampling and terrestrial predators [4], and by increasing structural heterogeneity, they can foster greater species richness and diversity than adjacent non-cliff habitats [1, 5, 6]. Furthermore, many species that use cliffs are adapted to cliff habitats and have elevated conservation concerns [7]. Thus, cliff habitats are unique, essentially non-renewable [8], havens for a wide range of species. Cliffs are understudied [2] and the lack of information about these ecosystems presents a challenge for land managers when determining how to regulate human disturbance on and around cliffs [4].

Historically, cliffs have been relatively undisturbed by humans during recreational activities, but the increased popularity of rock climbing could have novel impacts on these ecosystems. According to a survey by the Outdoor Foundation [9], 4.5 million Americans climbed at least once in 2012, and there is pressure on land managers to open new climbing areas in many parts of the country. It is well established that rock climbing can have detrimental effects on rock substrates, vegetation, and nesting birds of prey [10–13], but little is known about how communities of non-raptorial, cliff-specialist bird species respond to recreational climbing. The only study to investigate climbing impacts on avian communities suggested that rock climbing has negative impacts, primarily through altering the behavior and distribution of birds using cliff habitats [14]. Camp & Knight’s study [14], conducted in Joshua Tree National Park (JTNP) in the Mojave Desert, California, USA, found that climbing areas experienced considerable disturbance from climbers as well as other recreationists, which promoted the presence of invasive and common bird species while reducing avian diversity. Given the localized nature of this study and unique habitat within Joshua Tree National Park, additional studies examining the effects of climbing on birds are warranted to assess broader trends. Additionally, examining abiotic cliff features in conjunction with human use patterns and non-avian biotic diversity will contribute substantially to our general understanding of potential rock climbing impacts on avian cliff communities.

Our study examined the effects of rock climbing on cliff communities along the Front Range of Colorado on land managed by the city of Boulder’s Open Space and Mountain Parks (OSMP) department. Like many other land-management agencies, OSMP operates under a dual-purpose mandate of conserving habitat and providing recreational opportunities. OSMP supports diverse wildlife communities [15], and is a popular recreational destination. Boulder OSMP manages approximately 18,000 ha, and receives an estimated 6 million annual visits, of which 4% (240,000 individuals per year) engage in some form of recreational climbing. Indeed, it is considered a world-class climbing destination as the rock formations comprising “The Flatirons” were ranked eighth in a list of top climbing locations in the U.S. by XtremeSports [16]. Cliffs in this area provide essential nesting and foraging habitat for species including White-throated Swifts (*Aeronautes saxatalis*), Violet-green Swallows (*Tachycineta thalassina*), Rock Wrens (*Salpinctes obsoletus*), and Canyon Wrens (*Catherpes mexicanus*), which are the passerine species most tightly associated with cliff habitats in Colorado [7, 17]. The effects of recreational rock climbing on these (and other) cliff-dwelling species are unknown, but because some of them are typically present in low densities, human disturbance may impact territory occupancy and breeding success[18].

Although rock climbing is a popular recreational activity, the effects of this sport on avian cliff communities have not been thoroughly studied, and the effects of climbing on cliffs set within a forested matrix have never been studied. Our research addressed this knowledge gap by examining the overall avian community on and around cliffs in the flatirons climbing area, and assessing avian cliff use, cliff vegetation and arthropod communities. We compared cliffs with high and low levels of rock climbing activity and assessed how climbing level affected the following variables related to bird communities: avian diversity, avian species richness, and individual bird abundance on and around the cliffs. Additionally, we used Partners in Flight (PIF) species priority ranks as part of a formula developed by Nuttle et al. [19] to calculate avian community conservation value indices at each of our study sites [20]. This approach provides land managers with more precise data and assists with prioritizing conservation opportunities and decision-making processes. We incorporated both human recreational activity and physical attributes of cliff sites into models to assess which variables most strongly predicted measures of avian cliff communities.

An additional goal of our study was to estimate the cliff use patterns of our species of interest. For many cliff-associated birds, successful reproduction requires individuals to spend prolonged periods of time on cliffs either attending nests (e.g. white-throated swifts), or foraging (e.g. canyon wrens) [17, 21]. To determine use of cliffs by birds, we measured the amount of time they spent on cliffs during surveys. Finally, we surveyed arthropods and vegetation to investigate potential relationships between patterns of diversity and abundance among multiple taxa within our study area. We hypothesized that rock climbing activity would generally have negative effects on cliff communities, with high-use climbing formations having lower diversity of birds, arthropods and vegetation, lower relative abundances of birds, arthropods, and vegetation, and lower bird conservation value indices. We further predicted that birds would spend more time on cliffs that had low climbing activity.

## Methods

Permission was granted to conduct this research by the land management agency–City of Boulder Open Space and Mountain Parks Department. As the research was conducted on open space land and avoided wildlife closure areas, no specific permission was required to access any of the study sites. All surveys and sampling procedures were approved by OSMP. This research did not involve endangered or protected species. It also did not involve any handling of birds (or any other vertebrate species) and therefore no permit was required from either Colorado Parks and Wildlife or from the federal government. Observations of vertebrates were approved as part of UNCO IACUC protocol 1105c.

### Description of study area

**Fig 1:**
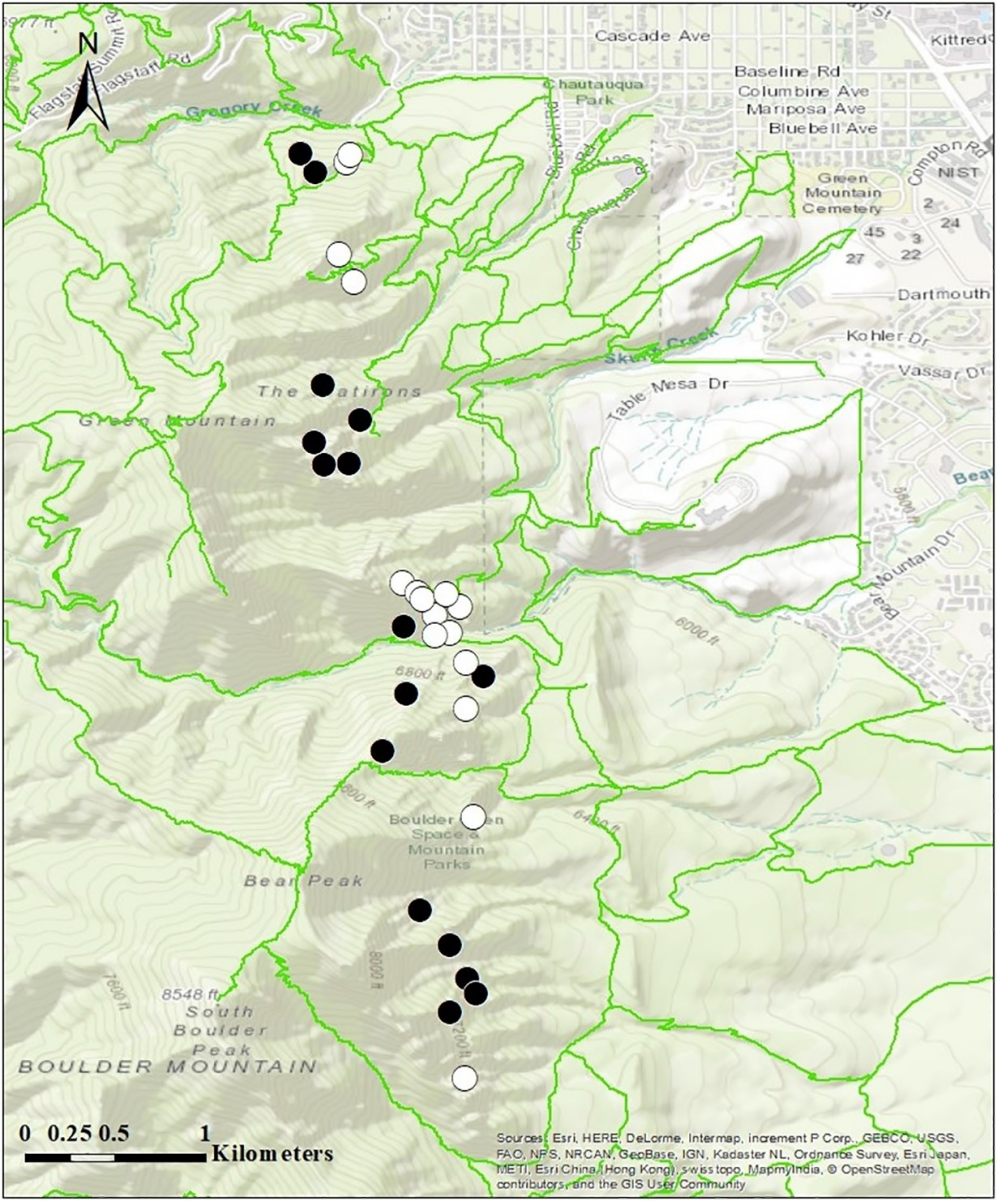
A map of the area where we studied cliff organisms west of Boulder, Colorado, USA, in 2015. Cliff formations used in the study are indicated by circles (black = low-use climbing sites, white = high-use climbing sites). Hiking trails are drawn in bright green. Basemap was created using ArcGIS software by Esri [25]. ArcGIS and ArcMap are the intellectual property of Esri and are used herein under license. Copyright Esri. All rights reserved. For more information about Esri software, please visit www.esri.com. Trails were obtained from the City of Boulder GIS portal [26].

Our study area in Boulder, Colorado, USA encompassed Gregory Amphitheater (39.9958°N, 105.2956°W) and the Flatirons northwest of Chautauqua Park south to the entrance of Shadow Canyon (39.9450°N, 105.2874°W) (Fig 1). This area falls within the Lower Montane Zone and has an elevation ranging from approximately 1,800 to 2,300 m above sea level. The Boulder Flatirons, composed of arkosic and conglomeratic sandstone, were created by the uplift and tilting of the Fountain Formation during the Laramide Orogeny [22]. Vertical angles of cliffs differ dramatically in relation to aspect as a result of the uplift. Cliffs facing west are likely to be slightly overhung, while those facing east have vertical angles ranging from 50–55° [23]. These structures form a broken line running north-south along already steep ridges [15]. Altogether, cliffs and talus comprise 9% of the land on OSMP [15]. The habitat surrounding the cliffs features large talus fields and forests of mixed Ponderosa Pine (*Pinus ponderosa*) and Douglas-fir (*Pseudotsuga menziesii*) [24].

### Study site selection

Study sites were selected from the rock formations available for bouldering and climbing within Boulder OSMP lands by considering climbing use ratings, locations of seasonal wildlife closures, and physical features of the cliffs. Climbing use ratings for each formation were provided by OSMP and were based on a 2011 poll conducted by the local climbing advocacy group, the Flatirons Climbing Council. The classifications for climbing use activity were as follows: *Low-* fewer than 100 visitors per year, *Intermediate-* between 100 and 500 visitors per year, and *High-* more than 500 visitors per year. Intermediate use cliffs were not included in this study. We compared avian diversity and behavior between 16 low and 16 high-use climbing cliffs, for a total of 32 study sites (Fig 1). Because nearly all large cliffs in the park are available for climbing, we were unable to compare climbing cliffs with no-use cliffs. A number of formations within the park are inaccessible from February1- July 31 to climbers, hikers, and researchers to protect nesting and roosting raptors. We excluded formations that were inaccessible due to wildlife closures.

To select sites, we initially matched high- and low-use formations by comparing aspect, height, vertical angle, and elevation, to ensure that physical cliff attributes had a wide range but were comparable between high and low use sites. Final study sites included cliffs with similar physical attributes (Table 1) and a nearly even distribution of aspects: 3 northward facing, 4 southward facing, 5 westward facing, and 4 eastward facing. Although there are established climbing routes on multiple sides of several formations, each formation was used as a study site from only one direction (either north, south, east, or west). We measured the heights of all potential climbing formations within OSMP using 0.75 m resolution LiDAR Digital Elevation Maps (DEMs) downloaded from the Colorado GeoData Cache [27] in ArcGIS. Cliff heights were calculated by measuring the elevation at the base of the cliff at the survey location and subtracting this from the maximum elevation of the formation at the survey location. Verticality was measured in the field using a clinometer. Information on the number of climbing routes was obtained from *Climbing Boulder’s Flatirons* [28]. Additional measurements of distances from each study site to streams [29], trails [26] and parking lots [30] were conducted in ArcGIS.

**Table 1:** Values of cliff attributes for study sites. Data are presented as averages ± standard error of the mean, *n* = 16 for each cliff category.

Cliff Attribute	High-Use Climbing Sites	Low-Use Climbing Sites
Height (m)	41.31±3.7	43.56±5.4
Vertical angle (degrees)	82.9±4	77.9±4
Elevation (m)	2,043.9±25	2,135.8±29
Total Routes on Formation	14.0±1.3	3.6±0.6
Routes on Surveyed Face	4.5±.7	1.8±0.4

### Avian observations

We conducted surveys of birds from May 10 to July 24, 2015 to assess species diversity and behavior. Each survey was one hour long. To account for variations in activity for both birds and climbers, we surveyed each site twice during early morning (n = 64 surveys) and mid-day (n = 64 surveys), and at least once during the evening (n = 54 survey) for a total of 182 surveys. Early morning included sunrise and the following three hours, mid-day was from 1030 to 1330, and evening was three hours prior to sunset. The order in which sites were visited was randomized for the first set of surveys. Sites were revisited in the same order for subsequent surveys.

**Fig 2:**
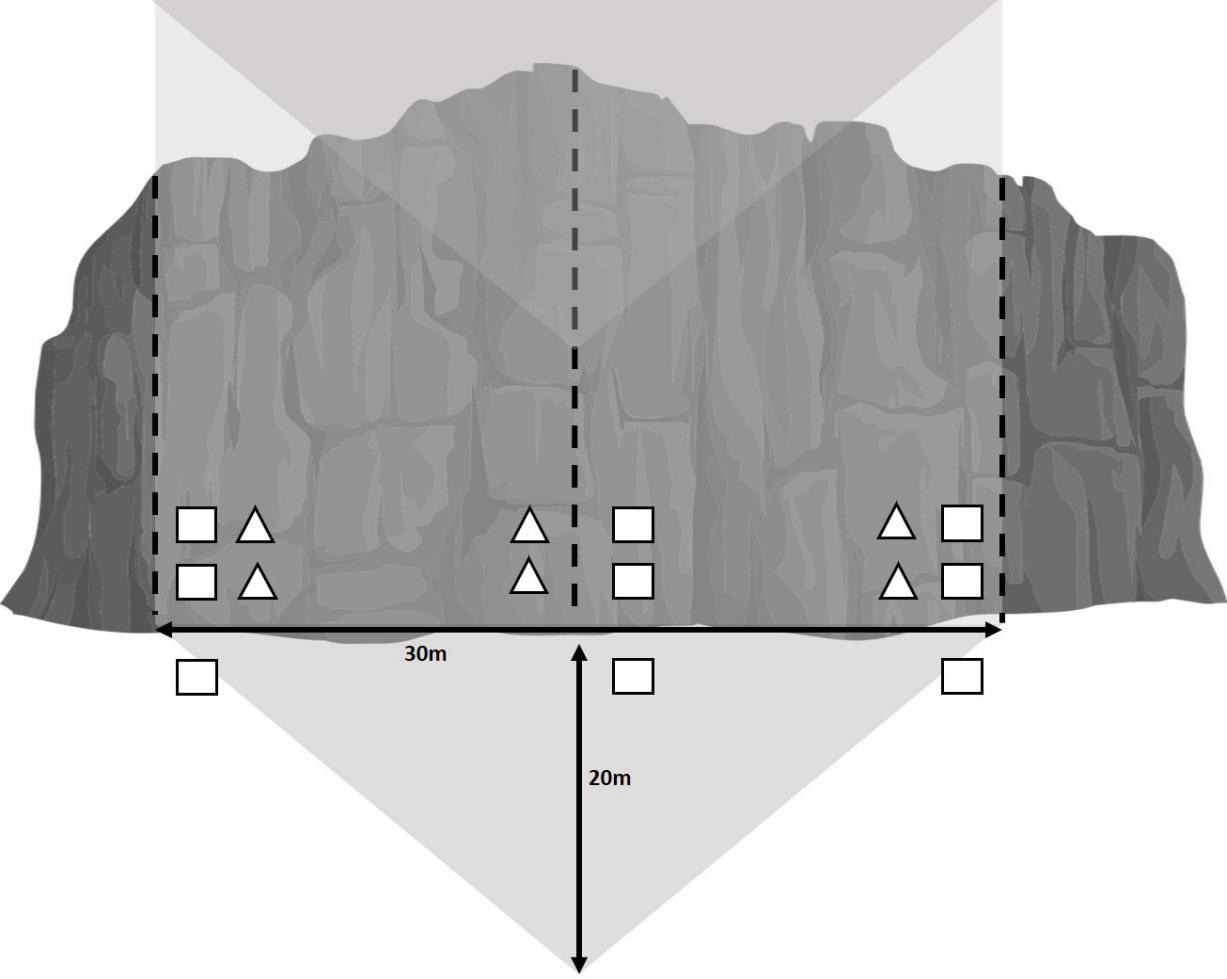
Diagram of survey area. The surveyed area is indicated in light gray and included a 30 m wide section of cliff (height varied with site), a triangular space stretching to the researcher positioned 20 m away from the cliff base, and the air space above the cliff and triangular space. Dashed lines indicate vertical vegetation transects, squares indicate 0.25 m^2^ vegetation plots, triangles indicate arthropod traps.

During the surveys, the researchers sat 20 m away from the base of the cliff to conduct observations of a 30 m wide section of cliff face (Fig 2). The relatively small size of this survey area was due to limitations in visibility in the Flatirons area given the forest matrix surrounding cliffs. The cliff face, as well as the space between the surveyor and the cliff and the air space immediately above this location, were included in the survey area (Fig 2). At one minute intervals the surveyor recorded the bird species present, the maximum number of individuals of all species observed (“bird abundance”), and all location(s) of the bird(s), differentiating bird’s use of the actual cliff versus the surrounding area. Additional data collected included the presence of climbers at the site.

### Vegetation surveys

We conducted vegetation surveys at a subset of our sites: 10 high climbing use and 10 low climbing use cliffs from July 10 to July 22. We quantified vegetative cover by estimating percent cover of vegetation types within 0.25 m^2^ plots, according to the PLANTS Database growth habit classifications [31]. This was done for ground cover 1 m away from the base of the cliff, cliff vegetation 1 m high on the cliff, and cliff vegetation 2.5 m high on the cliff (Fig 2). We used three replicates at every site for each of these categories: one at the approximate middle of the survey area and two at each end of the 30 m cliff section (Fig 2). For plots directly on the cliff face, we described lichen types as either crustose or foliose, and assigned a foliose:crustose ratio to determine the percent of foliose cover. As an additional metric of cliff-growing vegetation, we used 3 vertical cliff transects within each survey area and estimated the percent of the line that intersected vegetation (Fig 2). Estimates were based on observations from the ground. To further quantify vegetation near the base of the cliff we estimated the total number of trees greater than 3 m tall within the triangular study area.

### Arthropod sampling

At each of the 20 sites where vegetation surveys were conducted, we set out Trapper insect traps (Bell Laboratories, Inc) for 4 days in mid-July. The traps were folded paper triangles with scented glue at the base to attract and catch arthropods. These were attached to the cliffs by securing their bases with outdoor tape and lodging them into slight crevices in the rock. For each site, we set three traps less than 1m high on the cliff and three traps 2.5 m high on the cliff (Fig 2). Some of the traps fell from the cliffs while they were in the field and consequently were excluded from analysis; therefore the total number of traps included in analyses was 91, 44 from high-use climbing sites and 47 from low-use climbing sites. Traps were stored in a -20°C freezer upon retrieval. We identified all trapped specimens to taxonomic order and estimated biomass per trap according to the length-width calculations of [32].

### Statistical analyses

To test our hypotheses regarding the impacts of recreational rock climbing on cliff organismal communities, we evaluated the abundance and diversity of birds at all climbing sites using a model fitting approach. We used Linear Mixed Models (Standard Least Squares personality) and tested for model fit by examining the distributions of the residuals for normality. We included site (n = 32) as a random factor and the following as fixed factors: Climbing use (low/high; categorical), Climbers present (yes/no; categorical), Aspect (north/south/east/west; categorical), Cliff height (continuous), Distance to parking lots (continuous). Cliff height was included because it affected the size of the survey area, and distance to parking lots was included because it was shown to influence avian communities in the study conducted in Joshua Tree National Park [14]. In our survey area, there was collinearity between i) climbing use rating and number of routes on cliff, ii) aspect and vertical angle of cliffs, and iii) distance to streams, distance to trails, distance to parking lots, and elevation. Therefore, in each of these instances we included only the variable that we thought was most biologically relevant to avian communities (i.e. climbing use rating, aspect, and distance to parking lots respectively). We conducted a Backwards Stepwise Selection procedure based on Akaike’s Information Criterion corrected for small sample size (AIC_c_)[33]. We considered models with a ΔAIC_c_ within 2 of the best fit model to be significant and reported these in our results section. All avian surveys were included in these analyses.

Response variables included avian community conservation value (CCV), the number of scans in which we observed birds using the cliff (a measure of cliff use frequency), and the following metrics for both the full survey area and for the cliff face: species richness, numbers of individual birds, and Shannon-Wiener Diversity Index (*H’*) [34]. We calculated species richness values for each survey as the total number of species observed, and bird species abundance (both in the area and on the cliff) as the greatest number of individuals of a given species seen simultaneously during a single scan. CCV indices were representative of the entire survey area. We calculated these for each bird survey using the formula: CCV = S*Ʃ(RA_i_*w_*i*_) where S represents species richness for the survey, RA_*i*_ is the relative abundance of each species observed (total number of individuals of species *i* observed in survey/total number of individual birds observed in survey), and w_*i*_ is the weighted conservation score from 0 for non-native species to 4 for species of special concern [19], which is calculated based on Partners in Flight (PIF) 2007–08 Breeding Bird Survey data for Bird Conservation Regions 16 and 18 [20].

Arthropod measures included estimated biomass and *H’*, calculated using taxonomic orders. All arthropod traps at a single site were pooled and averaged for analyses. Vegetation measures included percent cover in ground plots and cliff plots, foliose:crustose ratio on cliff plots, percent cover on vertical transects, number of trees in the survey area, and *H’*, calculated using USDA growth types [31]. For measures of vegetative cover, vertical transects as well as ground, low cliff, and high cliff plots were pooled and averaged across each of the three vertical replicates at each site prior to inclusion in analyses. Because plant and arthropod data were collected at a subset of avian survey sites, were not sampled during avian surveys, and had only an n of 20, we did not include them in our linear mixed models. Instead, we used non-paired, non-parametric Mann-Whitney tests to evaluate whether high- and low-use climbing sites showed differences in arthropod biomass, vegetation diversity, and ratios of lichen types. All tests were one-tailed, unless otherwise noted because we hypothesized that high-use sites were more depauperate than low-use sites. We used sequential Bonferroni corrections to account for the multiple tests required for various measures of vegetation diversity and cover [35].

Lastly, we predicted there would be positive relationships between diversity and abundance of birds at cliff formations and diversity and abundance of other biota. We evaluated linear regressions of avian *H’* with *H’* of both arthropods and vegetation, and also evaluated correlations between the number of scans birds were observed on cliffs with both arthropod biomass and amount of vegetation cover (number of trees and percent cover in cliff and ground plots), as arthropods may influence foraging opportunities and vegetation cover may serve as a protective buffer. Regressions were run on site average data for the 20 sites at which we collected all data sets, 10 low- and 10 high-climbing use. All statistics were run in the program JMP, version 13.2. The values reported in the results section are mean ± SE. P-values of ≤0.05 were considered to be statistically significant, unless otherwise noted.

## Results

### Summary of avian observations

We completed a total of 182 avian surveys and observed 1,468 individual birds (although some of these could have been the same individual on different days) at our study sites. Total species richness was 45, with 37 species observed at high-use climbing sites and 39 species observed at low-use climbing sites (Table 2). Non-native species, such as European starlings (*Sturnus vulgaris*) and house sparrows (*Passer domesticus*), were never observed (Table 2). Species that were observed using the cliffs in our study area included White-throated Swifts, Violet-green Swallows, Common Ravens (*Corvus corax*), Prairie Falcons (*Falco mexicanus*), Peregrine Falcons (*Falco peregrinus*), Canyon Wrens, Townsend’s Solitaires (*Myadestes townsendi*), and occasionally White-breasted Nuthatches (*Sitta carolinensis*), and Rock Wrens. The other species observed during surveys were typically seen in the areas near or above the cliff. We observed climbers on the cliffs in 6.6% of the surveys comprising 38 individuals (11% of surveys and 35 individuals for high-use climbing sites and 2.2% of surveys and three individuals for low-use climbing sites). Although we did not observe high rates of climbing use during our study, our numbers are consistent with the categories supplied by the local climbing community, providing support for the validity of those categories.

**Table 2:** Numbers of high- and low-use climbing sites at which avian species were observed. Bold font indicates species which have previously been documented nesting in cliff habitats of Boulder Open Space and Mountain Parks [15].

Species	Scientific Name	PIF Rank^a^	Low-Use Climbing	High-Use Climbing
Double-crested Cormorant	*Phalacrocorax auritus*	2	0	1
**Turkey Vulture**	***Cathartes aura***	**2**	**15**	**14**
Cooper's Hawk	*Accipiter cooperii*	2	3	3
**Red-tailed Hawk**	***Buteo jamaicensis***	**2**	**1**	**1**
**Golden Eagle**	***Aquila chrysaetos***	**3**	**1**	**0**
Bald Eagle	*Haliaeetus leucocephalus*	3	1	0
**Mourning Dove**	***Zenaida macroura***	**2**	**1**	**1**
**White-throated Swift**^**b**^	***Aeronautes saxatalis***	**3**	**16**	**15**
Broad-tailed Hummingbird	*Selasphorus platycercus*	3	15	12
Rufous Hummingbird	*Selasphorus rufus*	3	1	0
Hairy Woodpecker	*Leuconotopicus villosus*	2	2	0
Northern Flicker	*Colaptes auratus*	2	3	3
**Peregrine Falcon**	***Falco peregrinus***	**2**	**1**	**1**
**Prairie Falcon**	***Falco mexicanus***	**4**	**3**	**2**
Cordilleran Flycatcher	*Empidonax occidentalis*	3	3	3
Hammond's Flycatcher	*Empidonax hammondii*	3	0	1
Say's Phoebe	*Sayornis saya*	3	0	1
Eastern Kingbird	*Tyrannus tyrannus*	2	1	4
Plumbeous Vireo	*Vireo plumbeus*	3	1	3
Warbling Vireo	*Vireo gilvus*	3	0	1
Stellar's Jay	*** ****Cyanocitta stelleri*	2	6	5
**Common Raven**	***Corvus corax***	**2**	**11**	**9**
**American Crow**	***Corvus brachyrhynchos***	**2**	**4**	**3**
**Tree Swallow**	***Tachycineta bicolor***	**2**	**1**	**0**
**Violet-green Swallow**^**b**^	***Tachycineta thalassina***	**2**	**16**	**16**
Mountain Chickadee	*Poecile gambeli*	2	10	5
Red-breasted Nuthatch	*** ****Sitta canadensis*	2	9	6
White-breasted Nuthatch	*** ****Sitta carolinensis*	2	13	8
Brown Creeper	*** ****Certhia americana*	2	1	1
House Wren	*Troglodytes aedon*	2	1	0
**Canyon Wren**	***Catherpes mexicanus***	**2**	**7**	**7**
**Rock Wren**	***Salpinctes obsoletus***	**2**	**3**	**2**
Kinglet species	*Regulus satrapa*,	2		
	*Regulus calendula*		1	0
Townsend's Solitaire	*Myadestes townsendi*	2	10	5
American Robin	*Turdus migratorius*	2	6	11
Virginia's Warbler	*Leiothlypis virginiae*	4	9	7
MacGillivray's Warbler	*Geothlypis tolmiei*	2	1	0
Yellow-rumped Warbler	*Setophaga coronata*	2	4	3
Yellow-breasted Chat	*Icteria virens*	2	0	1
Spotted Towhee	*** ****Pipilo maculatus*	2	2	3
Chipping Sparrow	*Spizella passerina*	2	2	1
Dark-eyed Junco	*Junco hyemalis*	2	6	6
Western Tanager	*Piranga ludoviciana*	2	7	6
Lazuli Bunting	*Passerina amoena*	3	0	1
Pine Siskin	*Spinus pinus*	2	6	2

^a^ PIF Rank is based on 2007–08 Breeding Bird Survey data for Bird Conservation Regions 16 and 18 and is on a scale of 0–4, with 4 indicating highest conservation priority and 0 used for non-native species.

^b^ Species observed nesting within our study areas in the 2015 field season.

**Fig 3:**
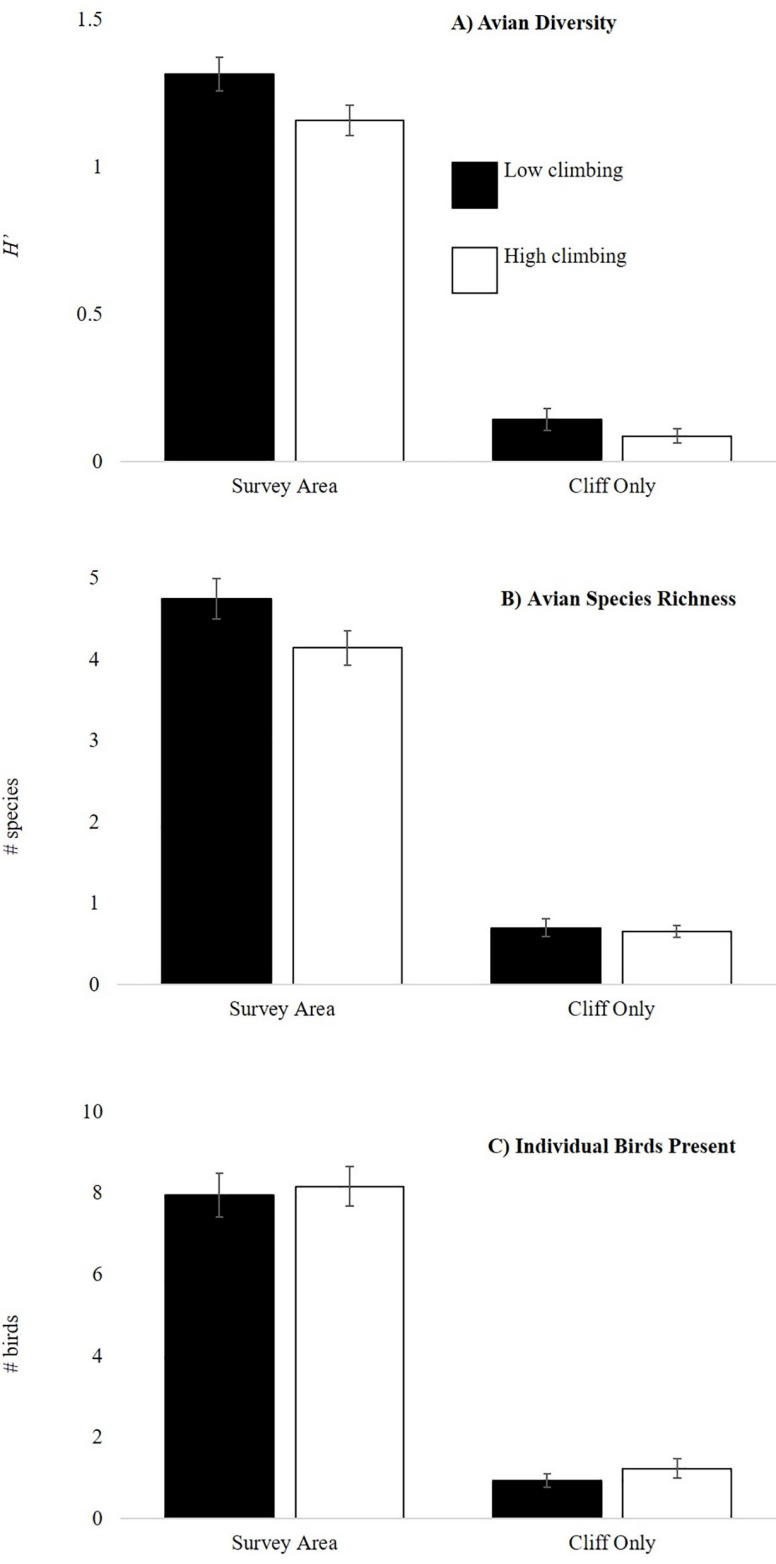
**Comparison of (A) avian diversity (B) avian species richness (C) number of individual birds present between high and low use climbing areas.** Numbers are based on overall survey averages for high and low-use climbing site surveys (*n* = 91each for the high and low categories) ± SEM. Individuals were summed across species. “Survey Area” refers to birds observed within the total survey area, “Cliff” indicates birds that were observed on the rock formation itself.

**Fig 4:**
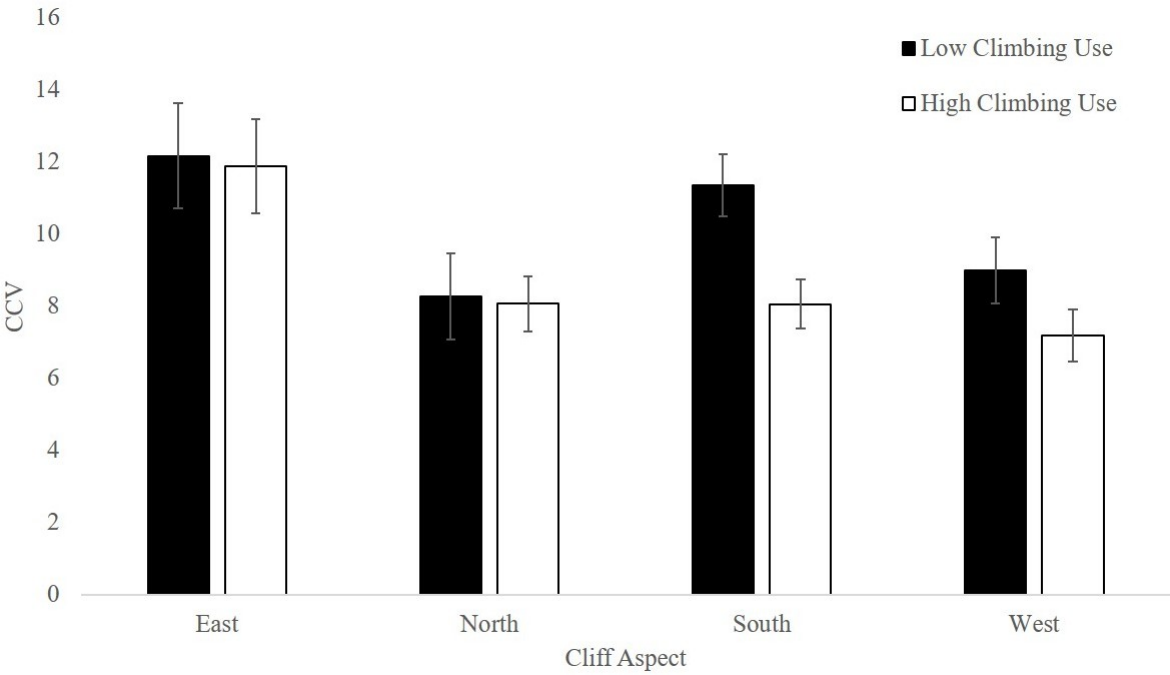
Community conservation value (CCV) by cliff aspect and climbing use rating. Numbers are based on overall survey averages for high and low-use climbing site surveys (n = 91 each for the high and low categories) ± SEM. Individuals were summed across species.

Avian diversity, species richness, and community conservation value (CCV) were higher within survey areas at low climbing use sites (Fig 3, Fig 4), however this difference was reduced on the cliffs relative to the whole survey area (Fig 3). Bird abundance was similar at high- and low-use climbing sites (Fig 3). Surprisingly, the numbers of individual birds on cliffs and scans that birds were observed using cliffs was higher at high-use climbing formations (Fig 3). Overall, species diversity, species richness, and bird abundance on the cliffs was much lower than the same metrics for the whole survey area (Fig 3).

Combined, results of linear mixed models (LMMs) indicated that climber presence and cliff aspect most strongly influenced the abundance and diversity of birds in local cliff communities, as each of these were included in four of the eight best fit models for our response variables (Table 3, S1 Appendix). Climbing use rating also predicted CCV and the number of scans birds were observed on cliffs. Cliff height was only included in one model where the delta AICc was <2 and distance to parking lots did not appear in any of the selected models (Table 3, S1 Appendix). For our measures of avian diversity and abundance on the cliff itself, three out of four best fit models were the null models that included only the random factor (site identity).

**Table 3:** Summary of models from LMMs for avian diversity, avian species richness, bird abundance, community conservation value, and cliff use where ΔAICc is within 2 of the best fit model. The best model for each response variable is listed first. See appendix for complete summaries of all evaluated models.

	Fixed factors included in the model	Model R^2^	ΔAICc relative to best fit model
**Entire Survey Area**			
***H’***	Climber presence	.201	0
	*None*	.181	1.50
**Species Richness**	Cliff aspect	.190	0
	Climber presence, Cliff aspect	.195	0.18
	Climbing use, Climber presence, Cliff aspect	.191	1.72
**Bird Abundance**	Climber presence, Cliff aspect	.194	0
	Climbing use, Climber presence, Cliff aspect	.200	1.77
	Cliff aspect	.180	1.77
**Conservation Value**	Climbing use, Climber presence, Cliff aspect	.258	0
	Climber presence, Cliff aspect	.262	0.07
**On Cliff**			
***H’***	*None*	.299	0
**Species Richness**	*None*	.325	0
**Bird Abundance**	*None*	.259	0
**Cliff Use ^a^**	Climbing use, Climber presence, Aspect	.407	0
	Climbing use, Climber presence, Aspect, Height	.402	0.58
	Climbing use, Aspect	.408	0.69

^a^ Cliff use is a measure of the number of scans birds were observed on the cliff.

### Avian diversity

On average, west-facing cliffs showed a trend of lower avian diversity (*H’*) within the survey area compared to other aspects, while east-facing cliffs generally had the highest avian diversity (*H’*_East_ = 1.45 ± 0.10, *H’*_South_ = 1.30 ± 0.09, *H’*_North_ = 1.10 ± 0.05, *H’*_West_ = 1.08 ± 0.07). Despite this, the best fit model of avian diversity in the entire survey area identified climber presence as the only significant predictor (Table 3, Table A in S1 Appendix). *H’* was lower when climbers were present compared to when they were absent (*H’* climbers present = 1.02 ± 0.10, *H’* climbers absent = 1.25 ± 0.04). None of our parameters had a significant effect on diversity when restricting analyses to birds on the cliff itself (Table 3, Table B in S1 Appendix).

### Species Richness

Cliff aspect was the strongest predictor of avian species richness within the survey area (Table 3). Similar to the pattern observed with our avian diversity results, species richness was highest at east- and south-facing cliffs (No.Species_East_ = 5.67 ± 0.40, No.Species_South_ = 4.54 ± 0.27, No.Species_North_ = 3.88 ± 0.31, No.Species_West_ = 3.81 ± 0.26). Although the best fit model for species richness in the survey area included only aspect as a predictor (Table 3, Table C in S1 Appendix), there was little difference (ΔAICc < 2) between the cliff aspect only, cliff aspect + climber presence, and cliff aspect + climber presence + climbing use models, suggesting that climbing pressure may have impacts on species richness (Table 3, Table C in S1 Appendix). Species richness on the cliff was not predicted by any of our modeled variables (Table 3, Table D in S1 Appendix).

### Abundance

Bird abundance in the entire survey area was best predicted by a model that included both climber presence and cliff aspect (Table 3, Table E in S1 Appendix). There were three models for bird abundance in the full survey area with ΔAICc < 2 that included cliff aspect, climber presence, and climbing use. Although more individual birds were typically observed at high climbing use sites (Fig 3), we observed fewer birds during surveys when climbers were present (5.85 ± 3.11 birds per survey) compared to when climbers were absent (8.24 ± 4.94 birds per survey). As with our diversity measures, east facing cliffs supported the greatest number of birds (No.Individuals_East_ = 10.17 ± 0.89, No.Individuals_North_ = 7.94 ± 0.76, No.Individuals_South_ = 7.69 ± 0.58, No.Individuals_West_ = 6.93 ± 0.62).Bird abundance on the cliff was not predicted by any of our modeled variables (Table 3, Table F in S1 Appendix)

### Community Conservation Value (CCV)

The best fit model for CCV, which was calculated for the full survey area, included climbing use, cliff aspect, and climber presence., with low climbing use and east-facing cliffs having the highest CCVs (Table 3, Fig 4, Table G in S1 Appendix). CCV indices for low-use cliffs were 10.21 ± 0.56 and 8.67 ± 0.48 for high-use cliffs. CCV indices with climbers absent were 9.69 ± 0.388 and 6.10 ± 0.946 with climbers present. The second best model, which showed a very minor increase in AICc included only climber presence and cliff aspect (Table 3, Table G in S1 Appendix).

### Cliff use

The top models for cliff use, measured as the number of scans birds were observed on the cliff, included climbing use, cliff aspect, climber presence, and, in one model within ΔAICc = 2 of the best-fit model, cliff height (Table 3, Table H in S1 Appendix). Surprisingly, birds spent more time on heavily climbed cliffs. On average, birds at high-use climbing sites were on the cliff for 6.96 ±1.44 scans per survey, while birds at low-use climbing sites were on the cliff for only 4.18 ±1.16 scans per survey. When climbers were present birds were on the cliff for 8.92 ±4.04 scans per survey, but when climbers were absent birds were on the cliff for only 5.31 ±0.94 scans per survey. We observed birds the most on south-facing cliffs, followed by north-facing cliffs (No.Scans_South_ = 8.98 ± 2.49, No.Scans_North_ = 7.26 ± 2.47, No. Scans_West_ = 4.72 ± 1.31, No.Scans_East_ = 1.48 ± 0.48).

### Vegetation and arthropods

There was no significant difference between high- and low-use climbing sites in either diversity or percent total vegetation cover, calculated by combining percent cover for vertical transects and both cliff and ground plots (Table 4). However, low climbing use plots had more foliose lichen cover and more trees, though the difference in tree cover was not significant at an α-level adjusted for multiple comparisons (Table 4).

**Table 4:** Summary of vegetation data (mean ± standard error) at high versus low use climbing sites.

Vegetation Metric	*n*	Low-Use Climbing	High-Use Climbing	U Statistic	Adjusted α-level	*p*-value
Foliose:crustose Ratio^a^	20	0.71±0.19	0.16±0.10	-3.21	0.0125	0.0013*
Number of Trees^b^	20	12.50±1.95	6.10±0.99	-2.39	0.0167	0.0168
Vegetation Cover^c^	20	51.23±5.29	48.37±4.35	-0.42	0.025	0.6776
*H*’^d^	20	0.41±0.10	0.36±0.07	-0.08	0.05	0.9397

* Asterisks indicate significance for Mann-Whitney tests.

^a^ Lichen cover was calculated for plots on cliffs and foliose:crustose ratio indicates the percent of the total lichen cover that was categorized as foliose.

^b^ Number of trees represents total trees >3m high within the survey area.

^c^ Vegetative cover is given as a percent of combined vertical transects and cliff and ground plots.

^d^ Diversity (H’) was calculated for ground plots only.

Presence of arthropod orders was similar between high- and low-use sites (Table 5). Mean *H’* for low-use sites was 1.20 (±0.10) and 1.08 (±0.09) for high-use sites (Mann-Whitney U:, *U* = -0.79, *p* = 0.43). Average arthropod biomass did not differ between low-use sites (13.58 ±3.17g) and high-use sites (11.85 ±2.94g; Mann-Whitney U:, *U* = -0.34, *p* = 0.74).

**Table 5:** Comparison of arthropod order counts and presence at high- versus low-use climbing sites. Numbers indicate at how many sites each order was observed. n = 10 sites of each type with 44 traps at high-use climbing sites and 47 traps at low-use climbing sites.

Order	High-Use	Low-Use
*Isopoda*	2	0
*Polyxenida*	9	6
*Opilones*	7	6
*Acari*	10	10
*Araneae*	8	8
*Thysanura*	1	1
*Collembola*	10	10
*Ephemeroptera*	1	0
*Odonata*	0	1
*Orthoptera*	6	6
*Dictypotera*	0	1
*Psocoptera*	7	8
*Hemiptera*	6	7
*Thysanoptera*	1	4
*Neuroptera*	0	1
*Coleoptera*	7	9
*Diptera*	10	10
*Lepidoptera*	3	1
*Hymenoptera*	7	9

Correlation analyses indicated that neither arthropod diversity (*R*^2^ = 0.004, F_19_ = 0.080, *p* = 0.78) nor vegetation diversity (*R*^2^ = 0.04, F_19_ = 0.69, *p* = 0.42) were significant predictors of avian diversity. However, arthropod abundance did predict the number of scans in which birds were observed on cliffs. Avian cliff use was correlated with arthropod biomass (*R*^2^ = 0.25, F_19_ = 5.93, *p* = 0.026), but not percent vegetative cover (*R*^2^ = 0.087, F_59_ = 1.70, *p* = 0.21).

## Discussion

Our research suggests that in a cliff and ponderosa pine forest matrix with relatively high recreation rates, rock climbing has negative impacts on cliff bird community diversity and conservation value, and mixed effects on individuals. Encouragingly, although climber presence and high rock climbing use affected a site’s avian species diversity and community conservation value, one of the best predictors of local avian diversity and cliff use was a natural physical characteristic: cliff aspect. East-facing cliffs had the highest avian species diversity while west-facing cliffs had the lowest. Additionally, bird abundance was not related to increases in climbing use, indicating that certain cliff-associated species are relatively tolerant of human activity. Finally, we detected only native avian species, including some considered of conservation concern in Colorado [36], suggesting that our study sites consisted of high-quality habitat.

We observed 45 native bird species using cliff habitats within our study area, including 13 cliff-nesting species previously documented in the area [15]. Presence of local cliff-nesting species was comparable between high- and low-use climbing sites. We identified 19 orders of arthropods living on and within cliff surfaces, and we documented variation in lichen among sites that experience different levels of rock climbing disturbance. At our study sites, climbing activities appear to be relatively infrequent, but can consist of large group sizes and vary substantially between sites, offering the potential to disrupt specific locations differently.

### Avian diversity and community conservation value

We found partial support for our hypothesis that rock climbing negatively impacts avian communities in cliff habitats. Avian diversity and CCV indices were generally higher at low-use rock climbing sites compared to high-use rock climbing sites and were also higher during surveys when climbers were absent. Greater CCV scores at low-use climbing and climber-absent cliffs suggest that high levels of rock climbing activity reduce the presence of avian species of conservation concern in the area [19]. The difference in CCV scores is important because human activities, including both development and recreational activity in an area, have been found to decrease densities of sensitive and/or specialist native species [37] even if species richness and diversity are similar.

Our model-fitting approach for the entire survey area revealed that avian diversity, abundance, and CCV were best predicted by combinations of cliff aspect, climber presence, and climbing use rating. Species richness was moderately affected by climbing use, however the best fit model only included cliff aspect. None of our cliff-only models found a negative impact of climbing on diversity, species richness, or abundance. This could reflect the much smaller sample size of birds using the cliff or perhaps species that use cliffs regularly are less affected by climber activity. Our results indicate i) that climber intrusion has a measurable negative effect on avian communities in the area but not necessarily on birds using the cliff and ii) that this effect is comparable to the influence of a natural attribute of the habitat.

Low-use rock climbing sites tended to have more trees compared to high-use sites, which could influence species composition by increasing habitat heterogeneity. East-facing cliffs had the highest diversity and CCV indices of birds and were unique in several ways. East-facing cliffs had the lowest angles (range: 47–63°), thus they receive more sunlight and have more vegetation growing on the cliff face (N.C. pers. obs.). South-facing cliffs, which also receive more sunlight compared to north and west-facing cliffs, had the second highest avian diversity. We hypothesize that thermal benefits as well as differences in vegetation composition influence spatial bird diversity, as has been documented in other studies [38–40].

We found that patterns of bird abundance did not align with patterns of diversity. In agreement with previous research [14], we found no difference in bird abundance at high- and low-use climbing sites. In other studies, high bird abundance was maintained at climbing sites via shifts to generalist and non-native species within a community [14, 41, 42]. While we found differences in avian CCV between high- and low-use climbing sites, the absence of non-native, generalist species, such as European Starlings, Brown-headed Cowbirds (*Molothrus ater*), and House Sparrows, in our study is encouraging. Furthermore, high-use climbing sites supported as many individuals of native cliff adapted species as did low-use climbing sites, suggesting that disturbance at high-use climbing sites in our study area was low compared to other areas [14], or was mitigated by physical attributes of the cliffs. Our finding that distance to parking lots had no effect on any of our avian cliff community metricsfurther supports this conclusion. It is possible that proximity to major human access points did not predict cliff community attributes because all parking lots were far enough away from our climbing formations to preclude such an effect.

### Avian cliff use

Interestingly, our hypothesis that birds would use the cliff face more often at low-use climbing formations was not supported. High-use sites had a higher average number of scans in which birds were observed on the cliff. This contrasts with existing research which found that birds at popular climbing cliffs were more likely to be located farther from the cliff face while birds at unclimbed cliffs were more likely to be either closer to the cliff or perched on the cliff face [14]. Our results may not align with previous work because of differences in location, recreation intensity, avian community, habituation of species, or landscape effects [6, 43, 44]. Additionally, tolerance to human intrusion varies among avian species [45]. While some birds tolerate or even thrive in areas of high anthropogenic activity, others are more sensitive and will flush quickly upon disturbance and eventually abandon an area that is overly stressful [42]. We hypothesize there may be a greater number or at least a greater percentage of anthropogenic-tolerant avian species in Boulder OSMP compared to JTNP.

Alternatively, physical characteristics of the cliffs themselves could cause differential cliff use by birds between high- and low-use climbing sites. In support of this, we found that aspect was also one of the best predictors of avian cliff use, with birds most frequently observed on north and south-facing cliffs. Avian diversity was higher at east-facing cliffs, a result that was driven by a variety of species and may be related to increased habitat heterogeneity at east-facing cliff sites. Activity patterns, in contrast, may be influenced by just a few species. Indeed, much of the cliff activity came from White-throated Swifts and Violet-green Swallows. Large numbers of these two species perching and nesting on north and south facing cliffs led to higher activity levels despite greater species diversity at east-facing cliffs. It is possible that rock climbers and the swift and swallow species in our area prefer similar cliff features, or that swifts and swallows are more tolerant of humans because they have a larger conspecific group size [44], or that a predator refuge effect is occurring. Other researchers have hypothesized that some species may associate with or tolerate human presence in order to escape from their predators, which are more wary of humans [46, 47]. This may explain why birds used the cliffs significantly more often at high climbing use sites. If humans had no effect on bird cliff use and cliff quality was equal, then we would expect cliff use among climbing use categories to be equal as well. Our results suggest that for some bird species, there may be a benefit of associating with climbers. Notably, raptors, which are sensitive to anthropogenic disturbance [11, 48], were not observed more often at high-use climbing sites. Rock climbing presents a serious threat to these birds because climbers have the ability to access areas in close proximity to nests [49].

### Vegetation and arthropods

Overall, our hypothesis that high-use climbing sites would have reduced vegetative cover and diversity was not supported. We did, find that there were more trees at low-use climbing sites, but this trend was non-significant when corrected for multiple comparisons. Lichens, however, do appear to reflect climbing pressure; low-use sites had significantly more foliose lichen cover compared to high-use sites. Our results support other studies which have documented negative impacts of rock climbing on delicate foliose lichens accompanied by a simultaneous increase in crustose lichen cover at climbing sites [50–52]. As such, it provides evidence that our high-use climbing sites did in fact have greater climbing activity than low-use climbing sites, and thus may be subject to disturbances documented in other studies.

We found no difference in either the diversity or biomass of arthropods between low- and high-use climbing sites. However, it is likely that our methods did not capture the full range of arthropod diversity present near cliffs. Because we know of no other studies which have described effects of rock climbing on arthropod diversity, more extensive research examining arthropods inhabiting cliffs is warranted.

We did not find a relationship among bird, plant, and arthropod diversity across sites, suggesting that avian diversity does not depend on the diversity of plants or arthropods located on cliffs within these habitats. In contrast, the abundances of different taxa were related; we found that avian cliff use was positively correlated with arthropod biomass. It’s possible that increased invertebrate prey at cliff sites may encourage birds to spend more time at those locations, although it should be noted that two of the commonly observed cliff specialist bird species were aerial insectivores (cliff swallows and white-throated swifts). Because our vegetation and arthropod surveys were done at a limited set of sites, and our collection and identification methods were conservative, we consider these results to be preliminary, and we encourage future study that more completely relates these community factors.

### Conclusion

Given our findings, we recommend that land managers combine analyses of human activity with information on habitat variation and species presence to determine which areas may be most affected by recreation. Our model suggests bird communities on north-facing cliffs were less diverse than bird communities on east-facing cliffs, but both of these were minimally affected by rock climbing, while communities on south- and west-facing cliffs were more impacted by human recreation (Fig 4). New climbing routes established on north-facing cliffs may cause less of a disturbance to a relatively lower number of bird species than new climbing routes on other cliff aspects. Furthermore, at least within our system, maintaining areas of high avian cliff use would serve to protect high arthropod and foliose lichen biomass.

The results of our study provide insights into cliff communities and how the organisms associated with them respond to rock climbing. Ecosystem responses may also be influenced by local conditions including dominant vegetation type, climate, landscape topography, and climbing intensity. Therefore, we recommend that more comprehensive studies of climbing impacts, including effects on nesting success, are initiated in different locations, and that they consider the combined influences of natural and anthropogenic factors.

## Supporting information

S1 Appendix Table A. Results of LMM for avian diversity (*H’*) for entire survey area.
Table B. Results of LMM for avian diversity (*H’*) on the cliff.
Table C. Results of LMM for avian species richness for entire survey area.
Table D. Results of LMM for avian species richness on the cliff.
Table E. Results of LMM for number of individual birds in entire survey area.
Table F. Results of LMM for number of individual birds on the cliff.
Table G. Results of LMM for CCV in entire survey area.
Table H. Results of LMM for number of scans with birds on the cliff.
(DOCX)
