# Supplementary material for: Rock climbing activity and physical habitat attributes impact avian community diversity in cliff environments
Source: PLoS One. 2019 Jan 16;14(1):e0209557. doi: 10.1371/journal.pone.0209557 (PMC6334907; doi:10.1371/journal.pone.0209557)
Supplement: S1 Appendix — Table A. Results of LMM for avian diversity (H’) for entire survey area. Table B. Results of LMM for avian diversity (H’) on the cliff. Table C. Results of LMM for avian species richness for entire survey area. Table D. Results of LMM for avian species richness on the cliff. Table E. Results of LMM for number of individual birds in entire survey area. Table F. Results of LMM for number of individual birds on the cliff. Table G. Results of LMM for CCV in entire survey area. Table H. Results of LMM for number of scans with birds on the cliff. (DOCX) [file pone.0209557.s001.docx]

**S1 Appendix**

The tables below contain the full results of linear mixed models for each of the avian response variables in our study. Bold text indicates the best model.

Models were constructed in JMP 13.2 using the Standard Least Squares personality. Each model included site (n=32) as a random factor and the following fixed factors: Climbing use (low/high; categorical), Climbers present (yes/no; categorical), Aspect (north/south/east/west; categorical), Cliff height (continuous), Distance to parking lots (continuous).

**S1 Table A.** **Results of LMM for avian diversity (*H’*) for entire survey area.**

| Climbing use [Low] | Climbers present? [N] | Aspect [East] | Aspect [North] | Aspect [South] | Height | Dist to parking | Model R^2^ | AICc | ΔAICc |
| --- | --- | --- | --- | --- | --- | --- | --- | --- | --- |
| 0.041 ± 0.046 | 0.18 ± 0.076 | 0.41 ± 0.14 | -0.19 ± 0.095 | 0.028 ± 0.072 | -0.0044 ± 0.0025 | 0.000030 ± 0.000067 | 0.197 | 328.46 | 40.99 |
| 0.050 ±0.041 | 0.18 ± 0.076 | 0.39 ± 0.12 | -0.17 ± 0.088 | 0.032 ± 0.07 | -0.0039 ± 0.0022 |  | 0.190 | 309.02 | 21.55 |
| 0.055 ± 0.042 | 0.16 ± 0.076 | 0.21 ± 0.074 | -0.10 ± 0081 | 0.040 ± 0.072 |  |  | 0.192 | 299.41 | 11.94 |
|  | 0.18 ± 0.075 | 0.21 ± 0.075 | -0.10 ± 0.083 | 0.038 ± 0.074 |  |  | 0.193 | 294.44 | 6.97 |
|  | **0.20 ± 0.075** |  |  |  |  |  | **0.201** | **287.47** | **0** |
|  |  |  |  |  |  | Null: | 0.181 | 288.97 | 1.50 |

Values in the first 6 columns are β ± standard error. Null model includes only formation as a random factor. ΔAICc = difference between the model’s AICc and the lowest AICc value

**S1 Table B.** **Results of LMM for avian diversity (*H’*) on the cliff.**

| Climbing use [Low] | Climbers present? [N] | Aspect [East] | Aspect [North] | Aspect [South] | Height | Dist to parking | Model R^2^ | AICc | ΔAICc |
| --- | --- | --- | --- | --- | --- | --- | --- | --- | --- |
| 0.018 ± 0.034 | -0.014 ± 0.042 | -0.017 ± 0.099 | 0.026 ± 0.070 | 0.042 ± 0.053 | 0.0029 ± 0.0018 | 0.000046 ± 0.000049 | 0.302 | 127.61 | 59.01 |
| 0.031 ± 0.031 | -0.013 ± 0.042 | -0.206 ± 0.092 | 0.049 ± 0.066 | 0.049 ± 0.053 | 0.0036 ± 0.0017 |  | 0.302 | 108.22 | 39.62 |
| 0.026 ± 0.033 | -0.0028 ± 0.042 | -0.043 ± 0.057 | -0.015 ± 0.063 | 0.042 ± 0.057 |  |  | 0.308 | 99.56 | 30.96 |
| 0.025 ± 0.032 |  | -0.043 ± 0.057 | -0.014 ± 0.062 | 0.042 ± 0.056 |  |  | 0.308 | 92.87 | 24.27 |
| 0.025 ± 0.031 |  |  |  |  |  |  | 0.300 | 75.12 | 6.52 |
|  |  |  |  |  |  | **Null:** | **0.299** | **68.60** | **0** |

Values in the first 6 columns are β ± standard error. Null model includes only formation as a random factor. ΔAICc = difference between the model’s AICc and the lowest AICc value

**S1 Table C.** **Results of LMM for avian species richness for entire survey area.**

| Climbing use [Low] | Climbers present? [N] | Aspect [East] | Aspect [North] | Aspect [South] | Height | Dist to parking | Model R^2^ | AICc | ΔAICc |
| --- | --- | --- | --- | --- | --- | --- | --- | --- | --- |
| 0.21 ± 0.19 | 0.47 ± 0.32 | 1.78 ± 0.57 | -0.77 ± 0.40 | -0.042 ± 0.30 | -0.013 ± 0.010 | 0.00013 ± 0.00028 | 0.199 | 826.15 | 26.52 |
| 0.25 ± 0.17 | 0.48 ± 0.32 | 1.68 ± 0.51 | -0.71 ± 0.37 | -0.021 ± 0.29 | -0.011 ± 0.0093 |  | 0.192 | 809.59 | 9.96 |
| 0.26 ± 0.17 | 0.43 ± 0.32 | 1.17 ± 0.30 | -0.51 ± 0.33 | 0.0016 ± 0.29 |  |  | 0.191 | 801.345 | 1.715 |
|  | 0.49 ± 0.31 | 1.16 ± 0.31 | -0.50 ± 0.34 | -0.0078 ± 0.30 |  |  | 0.195 | 799.81 | 0.18 |
|  |  | **1.19 ± 0.31** | **-0.59 ± 0.34** | **0.069 ± 0.30** |  |  | **0.190** | **799.63** | **0** |
|  |  |  |  |  |  | Null: | 0.211 | 806.21 | 6.58 |

Values in the first 6 columns are β ± standard error. Null model includes only formation as a random factor. ΔAICc = difference between the model’s AICc and the lowest AICc value

**S1 Table D.** **Results of LMM for avian species richness on the cliff.**

| Climbing use [Low] | Climbers present? [N] | Aspect [East] | Aspect [North] | Aspect [South] | Height | Dist to parking | Model R^2^ | AICc | ΔAICc |
| --- | --- | --- | --- | --- | --- | --- | --- | --- | --- |
| 0.0065 ± 0.099 | -0.12 ± 0.12 | -0.62 ± 0.29 | 0.083 ± 0.21 | 0.20 ± 0.16 | 0.0089 ± 0.0054 | 0.00011 ± 0.00015 | 0.330 | 494.80 | 42.53 |
| 0.039 ± 0.090 | -0.11 ± 0.12 | -0.71 ± 0.27 | 0.14 ± 0.19 | 0.22 ± 0.15 | 0.011 ± 0.0049 |  | 0.328 | 477.32 | 25.05 |
| 0.023 ± 0.096 | -0.084 ± 0.12 | -0.23 ± 0.17 | -0.047 ± 0.18 | 0.20 ± 0.17 |  |  | 0.333 | 470.81 | 18.54 |
|  | -0.082 ± 0.12 | -0.23 ± 0.16 | -0.047 ± 0.18 | 0.20 ± 0.16 |  |  | 0.331 | 465.80 | 13.53 |
|  | -0.073 ± 0.12 |  |  |  |  |  | 0.326 | 456.41 | 4.14 |
|  |  |  |  |  |  | **Null:** | **0.325** | **452.27** | **0** |

Values in the first 6 columns are β ± standard error. Null model includes only formation as a random factor. ΔAICc = difference between the model’s AICc and the lowest AICc value

**S1 Table E.** **Results of LMM for number of individual birds in entire survey area.**

| Climbing use [Low] | Climbers present? [N] | Aspect [East] | Aspect [North] | Aspect [South] | Height | Dist to parking | Model R^2^ | AICc | ΔAICc |
| --- | --- | --- | --- | --- | --- | --- | --- | --- | --- |
| -0.20 ± 0.48 | 1.13 ± 0.72 | 0.79 ± 1.43 | 0.42 ± 1.00 | -0.64 ± 0.76 | 0.025 ± 0.026 | 0.000036 ± 0.00071 | 0.208 | 1111.51 | 23.4 |
| -0.19 ± 0.43 | 1.13 ± 0.72 | 0.76 ± 1.29 | 0.44 ± 0.92 | -0.63 ± 0.74 | 0.026 ± 0.023 |  | 0.202 | 1096.57 | 8.46 |
| -0.23 ± 0.43 | 1.22 ± 0.72 | 1.91 ± 0.76 | -0.0058 ± 0.83 | -0.68 ± 0.74 |  |  | 0.200 | 1089.88 | 1.77 |
|  | **1.18 ± 0.71** | **1.91 ± 0.74** | **-0.015 ± 0.82** | **-0.67 ± 0.73** |  |  | **0.194** | **1088.11** | **0** |
|  |  | 1.98 ± 0.75 | -0.24 + 0.81 | -0.49 ± 0.72 |  |  | 0.180 | 1089.88 | 1.77 |
|  |  |  |  |  |  | Null: | 0.185 | 1094.68 | 6.57 |

Values in the first 6 columns are β ± standard error. Null model includes only formation as a random factor. ΔAICc = difference between the model’s AICc and the lowest AICc value

**S1 Table F. Results of LMM for number of individual birds on the cliff.**

| Climbing use [Low] | Climbers present? [N] | Aspect [East] | Aspect [North] | Aspect [South] | Height | Dist to parking | Model R^2^ | AICc | ΔAICc |
| --- | --- | --- | --- | --- | --- | --- | --- | --- | --- |
| -0.14 ± 0.22 | -0.049 ± 0.28 | -1.37 ± 0.64 | 0.68 ± 0.45 | 0.24 ± 0.34 | 0.017 ± 0.011 | 0.000046 ± 0.00035 | 0.267 | 789.04 | 33.53 |
| -0.13 ± 0.19 | -0.054 ± 0.28 | -1.41 ± 0.58 | 0.71 ± 0.41 | 0.25 ± 0.33 | 0.019 ± 0.010 |  | 0.263 | 772.51 | 17 |
| -0.16 0.20 | 0.011 ± 0.28 | -0.57 0.35 | 0.38 ± 0.39 | 0.21 ± 0.35 |  |  | 0.268 | 766.07 | 10.56 |
| -0.15 ± 0.19 |  | -0.57 ± 0.35 | 0.38 ± 0.38 | 0.22 ± 0.34 |  |  | 0.268 | 763.17 | 7.66 |
| -0.15 ± 0.20 |  |  |  |  |  |  | 0.262 | 758.39 | 2.88 |
|  |  |  |  |  |  | **Null:** | **0.259** | **755.51** | **0** |

Values in the first 6 columns are β ± standard error. Null model includes only formation as a random factor. ΔAICc = difference between the model’s AICc and the lowest AICc value

**S1 Table G.** **Results of LMM for CCV in entire survey area.**

| Climbing use [Low] |  | Climbers present? [N] | Aspect [East] | Aspect [North] | Aspect [South] | Height | Dist to parking | Model R^2^ | AICc | ΔAICc |
| --- | --- | --- | --- | --- | --- | --- | --- | --- | --- | --- |
| 0.520 ± 0.492 |  | 1.461 ± 0.719 | 3.357 ± 1.459 | -1.486 ± 1.025 | -0.0743 ±0.778 | -0.0194 ± 0.0267 | 0.000296 ± 0.000720 | 0.268 | 1109.77 | 22.19 |
| 0.608 ± 0.439 |  | 1.465 ± 0.717 | 3.121 ± 1.324 | -1.337 ± 0.946 | -0.0266 ± 0.758 | -0.0148 ± 0.0241 |  | 0.264 | 1095.05 | 7.47 |
| 0.632 ± 0.432 |  | **1.408 ± 0.711** | **2.457 ± 0.760** | **-1.078 ± 0.836** | **0.00161 ± 0.746** |  |  | **0.258** | **1087.58** | 0 |
|  |  | 1.539 ± 0.708 | 2.450 ± 0.781 | -1.056 ± 0.860 | -0.018 ± 0.767 |  |  | 0.262 | 1087.65 | 0.07 |
|  |  |  | 2.540 ± 0.448 | -1.353 ± 0.848 | 0.221 ± 0.759 |  |  | 0.237 | 1091.38 | 3.8 |
|  |  |  |  |  |  |  | Null: | 0.248 | 1100.21 | 12.63 |

Values in the first 6 columns are β ± standard error. Null model includes only formation as a random factor. ΔAICc = difference between the model’s AICc and the lowest AICc value

**S1 Table H.** **Results of LMM for number of scans with birds on the cliff.**

| Climbing use [Low] | Climbers present? [N] | Aspect [East] | Aspect [North] | Aspect [South] | Height | Dist to parking | Model R^2^ | AICc | ΔAICc |
| --- | --- | --- | --- | --- | --- | --- | --- | --- | --- |
| -2.20 ± 1.53 | -0.61 ± 1.67 | -8.91 ±4.52 | 2.71 ± 3.19 | 3.08 ± 2.44 | 0.11 ± 0.08 | 0.0036 ± 0.0022 | 0.418 | 1410.784 | 10.604 |
| -1.15 ± 1.35 | -0.63 ± 1.68 | -11.79 ± 4.05 | 4.55 ± 2.90 | 3.67 ± 2.34 | 0.17 ± 0.07 |  | 0.402 | 1400.76 | 0.58 |
| -1.41 ± 1.46 | **-0.15 ± 1.68** | **-4.14 ± 2.55** | **1.57 ± 2.82** | **3.35 ± 2.53** |  |  | **0.407** | **1400.18** | **0** |
| -1.42 ± 1.45 |  | -4.14 ± 2.55 | 1.60 ± 2.80 | 3.33 ± 2.52 |  |  | 0.408 | 1400.87 | 0.69 |
| -1.42 ± 1.47 |  |  |  |  |  |  | 0.405 | 1408.67 | 8.49 |
|  |  |  |  |  |  | Null: | 0.404 | 1410.13 | 9.95 |

Values in the first 6 columns are β ± standard error. Null model includes only formation as a random factor. ΔAICc = difference between the model’s AICc and the lowest AICc value
